# High Density Genetic Maps of Seashore Paspalum Using Genotyping-By-Sequencing and Their Relationship to The *Sorghum Bicolor* Genome

**DOI:** 10.1038/s41598-019-48257-3

**Published:** 2019-08-21

**Authors:** Peng Qi, Douglas Eudy, James C. Schnable, Jeremy Schmutz, Paul L. Raymer, Katrien M. Devos

**Affiliations:** 1 Institute of Plant Breeding, Genetics and Genomics (Department of Crop and Soil Sciences), University of Georgia, Athens, GA 30602 USA; 2 Department of Plant Biology, University of Georgia, Athens, GA 30602 USA; 3 Department of Agronomy and Horticulture, University of Nebraska, Lincoln, NE 68583 USA; 4 DOE Joint Genome Institute, Walnut Creek, CA 94598 USA; 5 HudsonAlpha Institute for Biotechnology, Huntsville, AL 35806 USA; 6 Institute of Plant Breeding, Genetics and Genomics (Department of Crop and Soil Sciences), University of Georgia, Griffin, GA 30223 USA; 7 Present Address: Monsanto Company, Huxley, IA 50124 USA

**Keywords:** Genetic linkage study, Next-generation sequencing, Plant genetics

## Abstract

As a step towards trait mapping in the halophyte seashore paspalum (*Paspalum vaginatum* Sw.), we developed an F_1_ mapping population from a cross between two genetically diverse and heterozygous accessions, 509022 and HI33. Progeny were genotyped using a genotyping-by-sequencing (GBS) approach and sequence reads were analyzed for single nucleotide polymorphisms (SNPs) using the UGbS-Flex pipeline. More markers were identified that segregated in the maternal parent (HA maps) compared to the paternal parent (AH maps), suggesting that 509022 had overall higher levels of heterozygosity than HI33. We also generated maps that consisted of markers that were heterozygous in both parents (HH maps). The AH, HA and HH maps each comprised more than 1000 markers. Markers formed 10 linkage groups, corresponding to the ten seashore paspalum chromosomes. Comparative analyses showed that each seashore paspalum chromosome was syntenic to and highly colinear with a single sorghum chromosome. Four inversions were identified, two of which were sorghum-specific while the other two were likely specific to seashore paspalum. These high-density maps are the first available genetic maps for seashore paspalum. The maps will provide a valuable tool for plant breeders and others in the *Paspalum* community to identify traits of interest, including salt tolerance.

## Introduction

Saline soils occupy some 2.1% of the world’s total land and as much as 19.5% of irrigated land (www.fao.org/soils-portal). Many crops are salt-sensitive and have reduced yields when grown in the presence of salt^1^. Consequently, there is significant interest to breed or genetically engineer crops with enhanced salt tolerance^2^. Furthermore, the quest for salt-tolerant crops will need to be accelerated over the next decades. The increased demand for food will necessitate expansion of crop cultivation into marginal areas that are already salt-affected or are vulnerable to salinization through seawater intrusion, storm surges and/or the salinizing effects of irrigation in arid areas^3^. With these future challenges in mind, we have initiated research to understand the tolerance mechanisms in the halophytic species seashore paspalum (*Paspalum vaginatum* Sw.). Seashore paspalum is able to survive exposure to even ocean-strength levels of salt, and as such has become an important turf grass in coastal and salt-affected areas of the world^4^. This panicoid grass is closely related to some of the world’s most important grain crops such as maize, sorghum and many of the millets, and may provide a gateway to improving these and other cereal crops for salt tolerance.

Seashore paspalum belongs to group Disticha, genus *Paspalum*, tribe *Paspalae* within the grass subfamily Panicoideae. The species is largely diploid (2*n* = 2*x* = 20) with a relatively small genome (C = ~600 Mb)^5^. Despite having been utilized as a turf for almost one hundred years, few genetic resources are available for seashore paspalum. A number of diversity analyses have been conducted that used random amplified polymorphic DNA (RAPD) markers^6^, amplified fragment length polymorphisms (AFLPs)^7^ and/or simple sequence repeat (SSR) markers^5,8–10^, but no genetic maps are available.

As a result of the revolution in molecular technologies, analyzing the genetic variation present in any plant germplasm, irrespective of its genome size and complexity, has become feasible^11^. In species with small and relatively non-complex genomes, whole genome (re)sequencing has become commonplace (*e.g*. rice, *Setaria*, tomato, soybean)^12–15^. For species with larger and/or more complex genomes (*e.g*. wheat, barley, maize, pearl millet, finger millet), where whole genome resequencing may still be cost prohibitive, reduced representation genome sequencing approaches such as restriction site associated DNA (RAD) sequencing and genotyping-by-sequencing (GBS) have paved the way for exploring genomic diversity and structure^16–19^. The utility of these approaches is not limited to species with high quality genome sequences, but extends to species such as seashore paspalum that have long suffered from a dearth of molecular resources. We employed GBS to develop several thousand single-nucleotide polymorphism (SNP) markers that segregated in an F_1_ population from a cross between *Paspalum vaginatum* accessions 509022 and HI33. These markers were used to construct the first genetic maps in the species. A comparative analysis between seashore paspalum and *Sorghum bicolor*, its closest relative with a fully assembled genome, showed a high level of colinearity between the two species.

## Materials and Methods

### Mapping population

An F_1_ mapping population comprised of 226 progeny was developed by crossing seashore paspalum accession 509022 with accession HI33. The origin of the lines is described in Eudy *et al*.^5^. The parental lines were chosen because they exhibited a large difference in their ranking for salt tolerance^20^. They were also genetically divergent. 509022 and HI33 belonged to two different genetic subpopulations as determined by an analysis with 43 SSR markers^5^.

### Tissue collection/DNA extraction

Healthy leaves were collected from the parents and mapping progeny, and flash frozen in liquid nitrogen. Samples were stored at −80 °C until processed further. Frozen leaves were ground in a TissueLyser II bead mill (Qiagen). DNA was extracted using the DNeasy Plant Mini Kit (Qiagen) following the manufacturer’s instructions, except that the volume of elution buffer (BufferAE) was reduced to 65 µL. Extracted DNA was quantified using a Nanodrop spectrophotometer and diluted to a working concentration of 50 ng/µL.

### GBS-library construction

DNA of the parents and progeny was digested with the restriction enzymes *Pst*I, *Nde*I and *Msp*I, and libraries were constructed as described in Qi *et al*.^19^. It should be noted that later experiments showed that this three-enzyme combination was not the optimal choice to get the maximum number of markers for the sequencing depth we obtained^19^. Two independent libraries were made for the maternal parent (509022) and three independent libraries were made from the paternal parent (HI33). Each library was individually quantified using the dsDNA HS (High Sensitivity) Assay Kit and a Qubit 2.0 Fluorometer (Invitrogen). A total of 45 ng of library from each of 184 progeny and from the parents were combined in a single pool. The pool of libraries was subjected to a double Solid Phase Reversible Immobilization (SPRI) selection at the Georgia Genomics and Bioinformatics Core (GGBC) to eliminate residual primers and DNA fragments outside the target range of 350–900 bps. The resulting DNA was sequenced on an Illumina NextSeq platform (paired-end 150 bps). GBS reads have been submitted to NCBI-SRA (Acc. PRJNA514362).

### Generation of a GBS reference and SNP calling

Generation of a GBS reference was conducted with the UGbS-Flex pipeline which combines different software packages with in-house perl and python scripts to generate a GBS reference from paired-end reads^19^. The parameters used in ‘ustacks’^21^ and ‘ASustacks’^19^ were ‘-m 2, -M 3 and –N 1’, and ‘-m 1, -M 3 and -N 1’, respectively, where ‘m’ is the minimum depth of coverage required to create a stack, ‘M’ is the maximum distance (in nucleotides) allowed between stacks and ‘N’ is the maximum distance allowed to align secondary reads to primary stacks. ‘Ustacks’ and ‘ASustacks’ cluster reads within and across accessions, respectively, based on the defined parameters, and identify a representative read for each cluster. We refer to this representative read identified by ‘ASustacks’ (across accessions) as a ‘GBS tag’. GBS tags derived from ‘ASustacks’ clusters that had representation from at least 50% of the accessions were selected to form the reference. If two or more tags had ≥95% sequence identity, only a single tag was included in the reference^19^. This reduced presence of allelic tags in the GBS reference.

Reads from each sample were aligned to either the GBS reference or to a highly fragmented seashore paspalum genome sequence assembly (J Schnable and J Schmutz, unpublished data) with Bowtie 2^22^, and SNP calling was done using Unified Genotyper from the Genome Analysis Toolkit (GATK)^23^. SNP filtering included removal of SNPs with three or more alleles, removal of SNPs with allele frequencies <0.1 and >0.9, and removal of adjacent SNPs, some of which had previously been shown to be artefacts derived from read misalignments^19^. SNPs with a read depth of at least 8X were converted to the mapping scores A, B, H, D (A or H) and C (B or H)^19^. SNPs within the same GBS tag were consolidated to a single marker as described by Qi and colleagues^19^. For SNPs identified against the *P. vaginatum* genome sequence, SNPs located within 500 bp of each other on the same scaffold were consolidated. Markers with less than 20% of missing data were used for genetic map construction.

### Creating maternal (HA), paternal (AH) and HH datasets

Based on the segregation ratios and the parental genotypes, the markers were divided into three sets. The ‘maternal dataset’ consisted of markers segregating only in the gametes contributed by the maternal parent (genotype H in accession 509022, and A or B in accession HI33). The ‘paternal dataset’ consisted of markers segregating only in the gametes contributed by the paternal parent (genotype A or B in 509022, and H in HI33). The HH dataset comprised the markers segregating in the gametes of both parents (both parents H). Maps generated with SNPs identified using the GBS data as reference were referred to as ‘GBS maps’ and those using the paspalum genome sequence were referred to as ‘Genome maps’. Chi-square tests were conducted to test markers for deviation from the expected 1:1 ratio (markers heterozygous in the maternal or paternal parent only) or 1:2:1 ratio (markers heterozygous in both parents). Markers with a p-value ≥ 1e-10 were selected for map construction. This threshold, which was determined empirically, provided a balance between removing markers with highly distorted segregation ratios that interfered with linkage group formation, and retaining chromosomal regions in the linkage map that carried factors affecting Mendelian segregation ratios. Markers that were heterozygous in one of the parents and appeared homozygous in the other parent, but segregated A, B and H across the progeny were discarded. The segregation ratios suggested that the apparent homozygous parental genotype carried a null allele and was, in fact, hemizygous (a- or b-).

Because we mapped in an outcrossing species, the linkage phase of the markers was unknown. Furthermore, whether an allele was designated as ‘A’ or ‘B’ depended on the allele present in the reference used for SNP calling. To make the data suitable for analysis in MAPMAKER, which does not have an algorithm to deal with outcrossing species, we modified the genotypic datasets. Please note that the modifications are different and have been described separately for HA/AH and for HH datasets. In the maternal (HA) and paternal (AH) datasets, ‘B’ scores in markers that were initially scored as ‘B’ in one parent and ‘H’ in the other parent were recoded to ‘A’. We then used the script ‘outcross-F1-AH-HA.py’ (http://research.franklin.uga.edu/devoslab/scripts-used-genetic-mapping) to duplicate the scores for each marker and reverse the marker scores in one copy of the duplicated dataset (‘H’ was changed to ‘A’, and ‘A’ was changed to ‘H’). Markers with reversed scores were identified with the suffix ‘r’ (exemplified in Table S1). The final dataset consisted of two different genotypic scores for each marker, and led to the formation of double the number of linkage groups expected based on the chromosome number. Pairs of linkage groups consisted of the same markers scored in different ways (Table S1). Only one linkage group per pair was used to generate a genetic map.

To group and order markers with contrasting linkage phase in the HH dataset using MAPMAKER, we modified the HH dataset using the script ‘outcross-F1-HH.py’ (http://research.franklin.uga.edu/devoslab/scripts-used-genetic-mapping). The modifications are outlined below and illustrated in Table S2. First, we duplicated the scores for each marker across progeny so that each marker had two scores per progeny. Then the entire marker set was duplicated as demonstrated in Supplementary Table S2. For one copy of the marker set, we kept the original scores in the first set of progenies, and changed ‘H’, ‘C’ and ‘D’ scores to missing data (‘−’), and ‘A’ and ‘B’ scores to ‘H’ in the second set of progenies. For the second copy of the marker set, we changed the scores as above in the first set of progenies and kept the original scores for the second set of progenies (Supplementary Table S2). These markers were given the suffix ‘d’ at the end of the marker name. We then duplicated the entire marker set again and changed ‘A’ scores to ‘B’ and *vice versa* in one of the copies (Supplementary Table S2). Markers with reversed ‘A’ and ‘B’ scores were given the suffix ‘r’ in the marker name. The final dataset consisted of four different genotypic scores for each marker. For markers that originated from a single chromosome, we expected linkage analysis to yield four linkage groups, each comprising the same markers scored in different ways (Supplementary Table S2). Marker ordering and genetic map construction was then conducted for one of the four ‘replicated’ linkage groups.

### Genetic map construction

Maternal, paternal and HH genetic maps were constructed using a modified version of MAPMAKER, essentially following the approach described by Qi *et al*.^19^. Population type was set as ‘backcross’ (BC) for the maternal and paternal data sets, and as ‘F2 intercross’ for the HH data set. Three progeny showed patterns consistent with sample contamination and were removed from the dataset. Where possible, map orders of markers that cosegregated were inferred from the physical positions of orthologous loci identified in the *Sorghum bicolor* (v2.0) genome. This was achieved by identifying the sorghum orthologs for each GBS tag as outlined under ‘Comparative Map Construction’, followed by manual reordering of cosegregating *Paspalum* markers based on their order in the sorghum genome. Map distances were calculated in centiMorgans (cM) using the Kosambi function.

### Comparative map construction

For SNP markers identified against the GBS reference, the GBS tags were used as queries in BLASTN searches using default parameters to the *Sorghum bicolor* genome v2.0^24^. For SNP markers identified against the paspalum genome sequence, regions spanning 500 bp on either side of the SNP were excised from the paspalum genome sequence and used as queries. For tags that returned hits at an e-value ≤ 1e-5, the first and second best hits were recorded. The top hits were used to determine comparative relationships. Markers that were located on orthologous chromosomes in seashore paspalum and sorghum were considered syntenic. Markers that were present in the same order on the orthologous chromosomes were defined as colinear. If the top hit did not fit the general pattern of observed synteny or colinearity, the second best hit was also considered in the comparative analysis. The locations of the top and, where relevant, secondary hits in the sorghum genome were plotted against the map positions of the SNP markers in seashore paspalum. The centromere locations annotated in the sorghum genome sequence were superimposed on the comparative maps to delineate the putative centromere locations in seashore paspalum.

## Results

### GBS-tags

The ‘ASustacks’ derived reference set, filtered to include tags present in at least 50% of the samples, contained a total of 13,184 sequence tags. Using these reference tags and after all filtering and consolidation steps, a total of 4078 SNP markers sequenced to a depth of ≥8X and with less than 20% of missing data was obtained for map construction. The number of markers for the HA, AH and HH maps was 1740, 1148 and 1190, respectively. When the highly fragmented seashore paspalum genome was used as reference for SNP calling, 1885 HA, 1654 AH and 1290 HH markers were obtained after filtering and consolidation.

### Paspalum genetic maps

**Figure 1:**
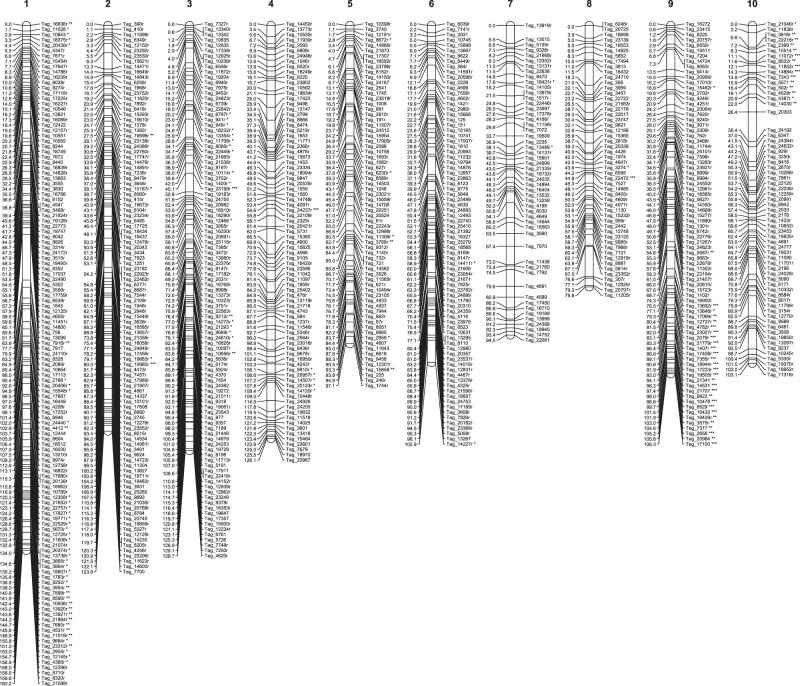
Maternal genetic map comprised of markers that were heterozygous in 509022 and homozygous in HI33. SNP markers were identified using a fragmented seashore paspalum genome sequence as reference. Only one marker per set of cosegregating markers is shown. Markers that deviated significantly from the Mendelian segregation ratio of 1:1 are indicated with * (deviation at the 5% level), ** (1% level) or *** (0.1% level) next to the marker name.

**Figure 2:**
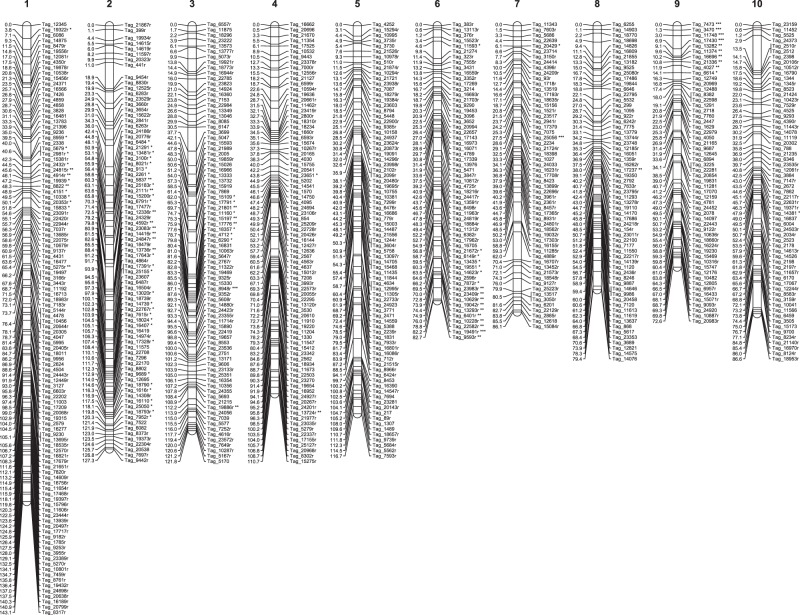
Paternal genetic map comprised of markers that were homozygous in 509022 and heterozygous in HI33. SNP markers were identified using a fragmented seashore paspalum genome sequence as reference. Only one marker per set of cosegregating markers is shown. Markers that deviated significantly from the Mendelian segregation ratio of 1:1 are indicated with * (deviation at the 5% level), ** (1% level) or *** (0.1% level) next to the marker name.

Seashore paspalum is largely self-incompatible and highly heterozygous and, hence, mapping was done using a pseudo-testcross approach in the F_1_ generation. For all maps generated, 10 linkage groups (LGs) were obtained, corresponding to the 10 seashore paspalum chromosomes. The number of markers and genetic length of each linkage group for each of the six maps are given in Table 1. The ‘Genome’ linkage maps are presented in Figs 1 (maternal map), 2 (paternal map) and 3 (HH map), and the ‘GBS’ maps are presented in Supplementary Figs S1 (maternal), S2 (paternal) and S3 (HH). For clarity, only one marker per group of cosegregating markers was included in the map charts. Full maps, including cosegregating markers, with genotypic scores are given in Supplementary Table S3. Sequence information for the mapped SNPs is given in Supplementary Table S4. Regions exhibiting highly significant segregation distortion (p ≤ 0.01) were found on LG 1, LG 9 and LG 10 in the maternal map (Fig. 1 and Supplementary Fig. S1), and on LG 1, LG 2, LG 3, LG 6 and LG 9 in the paternal map (Fig. 2 and Supplementary Fig. S2). Regions of severe segregation distortion were unique to each of the parental maps. Because of score duplications and conversions, segregation distortion was not calculated for the HH maps.

**Table 1:** Marker number and length for the generated genetic maps.

		GBS Reference			*P. vaginatum* Genome Reference		
		Maternal (HA)	Paternal (AH)	HH	Maternal (HA)	Paternal (AH)	HH
LG 1	Marker No.	284	192	160	287	296	191
	Map length (cM)	147	139	121	160	143	129
LG 2	Marker No.	248	127	181	269	177	161
	Map length (cM)	124	115	131	124	127	82
LG 3	Marker No.	236	94	158	249	185	146
	Map length (cM)	129	123	93	130	122	124
LG 4	Marker No.	184	125	92	198	179	119
	Map length (cM)	125	113	76	127	111	123
LG 5	Marker No.	154	141	83	196	177	95
	Map length (cM)	107	118	134	98	117	123
LG 6	Marker No.	194	95	106	215	125	128
	Map length (cM)	95	87	78	103	83	74
LG 7	Marker No.	81	92	91	102	124	115
	Map length (cM)	102	90	87	95	86	104
LG 8	Marker No.	89	58	95	93	110	100
	Map length (cM)	80	78	113	80	79	81
LG 9	Marker No.	155	86	93	161	111	107
	Map length (cM)	107	74	80	106	73	72
LG 10	Marker No.	115	138	131	115	170	128
	Map length (cM)	103	87	97	103	87	101
Total	Marker No.	1740	1148	1190	1885	1654	1290
	Map length (cM)	1119	1022	1010	1125	1028	1013

**Figure 3:**
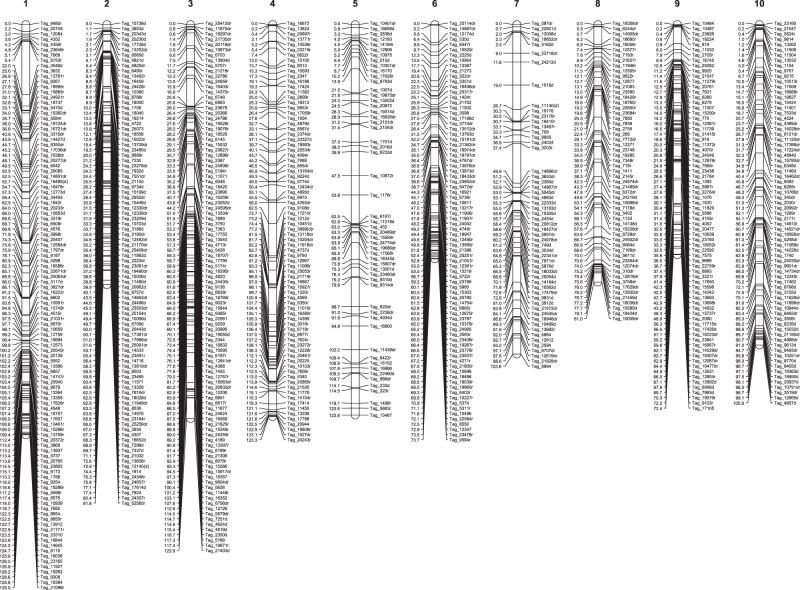
HH genetic map comprised of markers that were heterozygous in both 509022 and HI33. SNP markers were identified using a fragmented seashore paspalum genome sequence as reference. Only one marker per set of cosegregating markers is shown.

### Comparative relationships with sorghum

**Figure 4:**
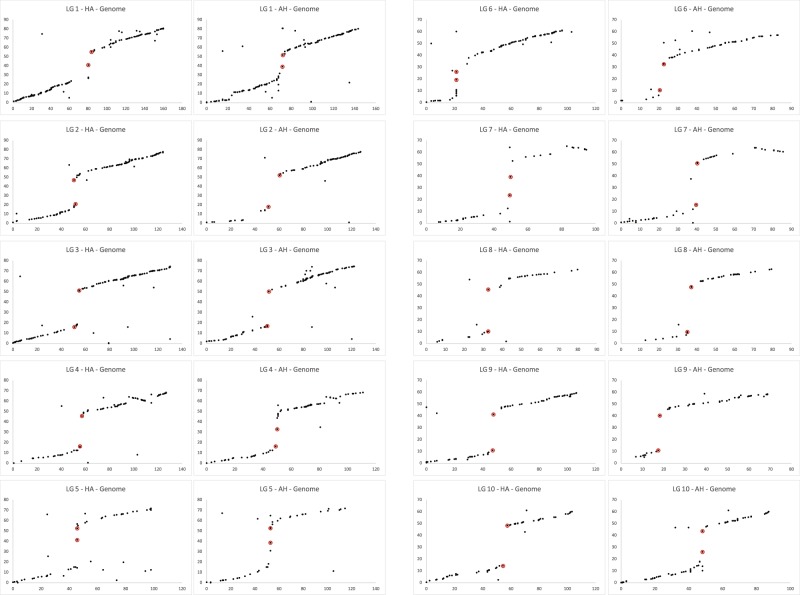
Dot plots showing the relationship between the seashore paspalum genetic maps (HA-Genome and AH-Genome) (X-axis) and the sorghum genome sequence (Y-axis). Markers flanking centromere locations in sorghum are indicated in red.

Of the 4078 GBS tags mapped, 2311 (56.7%) returned at least one hit in the *Sorghum bicolor* genome, the closest diploid species to seashore paspalum with a reference genome sequence, at an e-value threshold 1e-5. Comparative datapoints were obtained for 80.2% of the SNP markers identified against the *P. vaginatum* genome. The percentage of comparative markers that were in syntenic position was similar for the two references (GBS reference: 69.7%; genome reference: 67.3%). A comparison by linkage group across the six maps, however, showed that the percentage of markers with a putative syntenic ortholog in the sorghum genome was significantly lower for LG 5 than for the other linkage groups (37.2% for LG 5 *versus*, on average, 69.4% for the other LGs for the GBS reference; 37.4% for LG 5 *versus*, on average, 67.4% for the other LGs for the genome reference; Supplementary Table S5). LG 5 was the only linkage group for which the percentage syntenic markers deviated from the mean by more than 1.5 standard deviations in all six maps. The majority of syntenic markers mapped to colinear positions (GBS reference: 95.3%; genome reference: 92.5%). The number of comparative markers, syntenic markers and colinear markers per chromosome is given in Supplementary Table S5. While we recorded the top two blast hits, 98.5% of the colinear locations corresponded to the top hit. Dot plots and circos diagrams showing the comparative relationships of *P. vaginatum* with sorghum are presented in Fig. 4 (dot plots; maternal and paternal Genome maps) and in Supplementary Fig. S4 (dot plots and circos diagrams; all maps).

Each seashore paspalum linkage group largely corresponded to a single sorghum chromosome (Fig. 4 and Supplementary Fig. S4), and paspalum linkage groups were therefore oriented and numbered according to their synteny with sorghum. A known inversion in sorghum LG 4 spanning the region 57.81 to 64.36 Mb^25^ differentiated sorghum from all seashore paspalum LG 4 maps (maternal, paternal and HH). The number of comparative markers encompassing the inversion varied from three in the paternal GBS map to 26 in the maternal Genome map. A second inversion, also specific to sorghum, extended from position 59.58 Mb until the end of LG 7^25^. This inversion was also seen in all sorghum - paspalum comparative maps and was characterized by between five (maternal GBS map) and 14 comparative markers (paternal Genome map). Two small rearrangements on LG 1 (corresponding to the region 63.37–64.30 Mb in sorghum) and LG 5 (corresponding to the region 14.29–15.12 Mb in sorghum) had not previously been identified in any grass comparative analyses and, hence, were likely paspalum specific. The LG 1 inversion was identified in three of the six maps (AH Genome, HA GBS and AH GBS maps) by either three or four comparative markers. In the other three maps, the number of markers was insufficient to confirm the presence of this inversion. The LG 5 inversion was identified by four markers only in the HA Genome map. As for LG 1, this region on LG 5 was not covered by sufficient markers to determine the presence of a rearrangement in the other maps.

## Discussion

### Generation of HH maps in a pseudo-testcross population

A typical approach for linkage mapping in an obligate outcrossing species (F_1_ generation) is to generate maternal and paternal maps that capture recombination in the female and male parent, respectively, using a pseudo-testcross design. While software such as JoinMap^26^ and OneMap^27^ can integrate the parental maps with markers that are heterozygous in both parents, uncertainty about the linkage phase increases marker order ambiguity. Furthermore, we prefer to use MAPMAKER^19,28^ which has superior error handling ability^29^, so we opted to generate separate maternal and paternal maps. However, because the GBS technology yielded more than 1000 SNP markers that were heterozygous in both parents, we endeavored to also construct F_2_-type linkage maps. To ensure that markers that were derived from the same chromosome would link together irrespective of their linkage phase, we duplicated all genotypic scores across progenies (referred to as progeny sets 1 and 2) and across markers (referred to as marker sets 1 and 1d). We then converted the genotypic scores as exemplified in Supplementary Table S2. A second round of duplication and conversion (Supplementary Table S2) was carried out to account for the fact that the parental allele composition was unknown. This led to each chromosome being represented by four linkage groups, each consisting of the same mixture of markers from marker sets 1, 1d, 1r and 1dr. For example, if a linkage group consisted of ‘M1, M2d, M3r, M4dr’ with M1-4 being markers, the three corresponding linkage groups comprised markers ‘M1d, M2, M3dr, M4r’, ‘M1r, M2dr, M3, M4d’ and ‘M1dr, M2r, M3d, M4’. Marker ordering and genetic map construction was done for only one of the linkage groups. The colinear order of the comparative markers showed that this mapping approach resulted in valid maps. The rationale for generating maternal, paternal as well as HH maps was to allow future mapping of QTL for traits that were segregating only in the female parent, only in the male parent, or in both parents, respectively.

### GBS reference vs. genome reference

**Figure 5:**
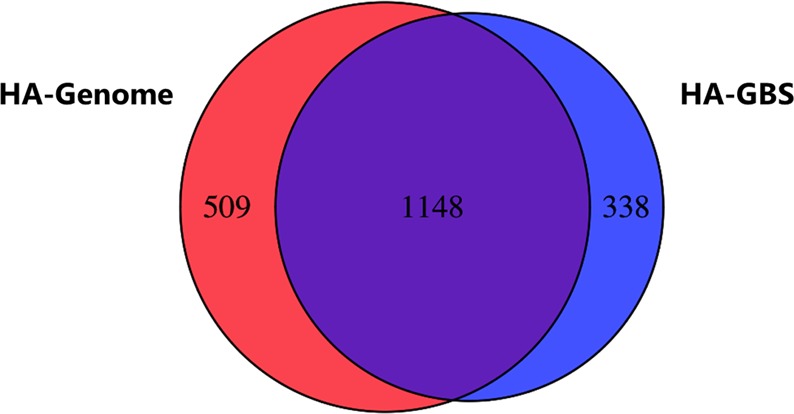
Venn diagram showing the number of scaffolds from the fragmented seashore paspalum reference genome assembly that were anchored to the HA Genome and HA GBS maps.

One of the advantages of GBS is that this methodology can be applied to species without a reference genome. Because not all GBS reads are assembled into reference tags, possibly because of allelic differences or sequencing errors, more SNPs will be identified when a whole-genome sequence assembly is used as reference compared to a GBS reference^30^. However, we wondered whether this was still true when the available reference genome sequence was highly fragmented. The seashore paspalum genome assembly available when we conducted our investigation consisted of a total of 117,840 scaffolds, only 1855 of which were larger than 50 kb (J. Schmutz and J. Schnable, unpublished data). Following comparable SNP filtering approaches, the number of markers identified using the fragmented genome sequence as reference was 8% higher for the maternal map and for the HH map, and 44% higher for the paternal map compared to using the GBS reference. Because of marker consolidation, we could not directly compare SNP markers identified with the two methods. We therefore determined the number of seashore paspalum scaffolds that could be anchored by the Genome maps and GBS maps. Anchoring to the GBS maps was done by BLASTN analysis (e-value ≤ 1e^−5^) of GBS tags corresponding to mapped SNPs against the seashore paspalum genome sequence. On average, 52% of the anchored scaffolds were common to both maps (Fig. 5 and Supplementary Fig. S5). Although 8% to 44% more SNP markers were mapped in the Genome maps than in the GBS maps, around 16% of the anchored scaffolds were secured to only the GBS maps. This can likely be explained by the fact that the genome sequence we used was an early draft from which not all allelic regions had been filtered out. The presence of two alleles in the reference used for aligning the GBS reads leads to the reads corresponding to each allele in a heterozygous individual being aligned to a different scaffold. Consequently, the two alleles are scored as monomorphic^19^.

When marker numbers were compared between the Genome and GBS maps per linkage group, there were significant differences between linkage groups, but these differences were not consistent across the maternal, paternal and HH maps. For example, the marker difference was highest (more than one standard deviation different from the mean) for LG 5 and LG 7 in the maternal map, for LG 3 and LG 8 in the paternal map, and for LG 2, LG 3, LG 4 and LG 7 in the HH map. Contrary to the overall pattern of lower marker numbers in the GBS maps than in the Genome maps, the reverse was observed for LG 2 and LG 3 in the HH maps.

Marker numbers in the maternal map were some 14% higher than in the paternal maps when the genome sequence was used as reference. This difference increased to 52% when the GBS reference was used. The overall lower number of markers in HI33 (paternal parent) compared to 509022 (maternal parent) in both the Genome map and the GBS map suggests that fewer loci were heterozygous in HI33 than in 509022. However, it is unclear why marker number differences between the maternal and paternal maps were considerably higher in the GBS maps compared to the Genome maps.

### Relationship between the seashore paspalum and sorghum genomes

The use of methylation-sensitive restriction enzymes for GBS ensured that the GBS tags were enriched for genic regions, and hence could be used for comparative analyses. Some 55% of GBS tags had hits in the closest sequenced relative, sorghum. We expect that the number of genic tags is actually higher because tags derived from promoter regions and introns would likely be too diverse to have homology across species. This is supported by the fact that 1 kb paspalum fragments, extracted from the paspalum genome sequence to span 500 bp on either side of mapped SNPs, were able to detect putative orthologs in sorghum at frequencies of, on average, 80.2%. When we reduced the region extracted from the paspalum genome sequence to 150 bp (75 bp on either side of a SNP), the percentage of query sequences that had blast hits (e-value ≤ 1e-5) in sorghum was reduced to 42.7%. Overall, a high level of gene order conservation was observed between seashore paspalum and sorghum. Each seashore paspalum chromosome corresponded to a single sorghum chromosome. Only four intrachromosomal inversions were identified. The two largest inversions (on LG 4 and LG 7) had previously been shown to have occurred in sorghum^25^. The two smaller putative inversions on LG 1 and LG 5 likely occurred in the seashore paspalum genome.

The comparative analyses showed almost complete coverage of the sorghum chromosomes by seashore paspalum markers (Supplementary Fig. S6). For most linkage groups, the most distal markers were located less than 5% from the ends of the sorghum chromosomes. Notable exceptions were LG 2 in the HH Genome map, which lacked ~18% of the short arm of the corresponding sorghum chromosome, LG 9 in the AH GBS map, which lacked ~17% of the short arm of the corresponding sorghum chromosome, and LG 6, which lacked the entire sorghum short arm in both the HH Genome and HH GBS maps. Sorghum chromosome 6 is acrocentric, and the majority of the short arm consists of pericentromeric heterochromatin^31^. A low frequency of loci that are heterozygous in both parents combined with the presence of fewer genic tags on this chromosome likely account for the absence of the short arm of LG 6 in the HH maps. GBS tags were almost completely absent from the centromeric regions in sorghum (Fig. 4 and Supplementary Fig. S4). This can be explained by (1) the majority of GBS tags being derived from genic regions and (2) repeat sequences being largely species-specific^32^. These marker-free regions, together with the centromere locations in sorghum were useful in determining the location of putative centromeres on the seashore paspalum genetic maps (Fig. 4, Supplementary Fig. S4 and Supplementary Table S3).

LG 5 had significantly fewer comparative data points than the remaining LGs. In earlier comparative analyses, fewer rice BACs could be anchored to sorghum chromosome 5 than to the other sorghum chromosomes^33^. We hypothesize that this orthology group contains a higher proportion of fast-evolving genes than other orthology groups. One such group of fast-evolving genes are disease resistance genes, which are overrepresented on both sorghum chromosome 5 (16% of annotated disease resistance genes^34^) and on its ortholog in rice, chromosome 11 (25% of annotated disease resistance genes^35^). We anticipate that paspalum LG 5 also carries a disproportionate number of disease resistance gene clusters.

## Conclusions

GBS made it possible to generate the first genetic maps of seashore paspalum, an obligate outcrossing species, comprising several thousand SNP markers. Three maps were generated for each linkage group that represented recombination events in the female parent, the male parent, and both parents. The markers were distributed genome-wide and enriched for the genic regions of the paspalum genome. The maps demonstrated that seashore paspalum is largely colinear with sorghum, despite representing the more distant Paspaleae clade. These genetic maps will be useful for future quantitative trait loci (QTL) mapping in seashore paspalum and improvement of the paspalum genome sequence. Furthermore, the comparative relationship will help to identify regions and, ultimately, genes that vary between seashore paspalum, a halophyte, and its glycophytic relatives such as sorghum, maize and *Setaria*.

## Data Availability

GBS reads have been submitted to NCBI-SRA (Acc. PRJNA514362). SNPs with flanking sequence and the genotypic scores for each SNP marker are provided as Supplementary Information.

## Supplementary information

Supplementary Figures 1 to 6

Table S1

Table S2

Table S3

Table S4

Table S5
