# Supplementary material for: High Density Genetic Maps of Seashore Paspalum Using Genotyping-By-Sequencing and Their Relationship to The Sorghum Bicolor Genome
Source: Sci Rep. 2019 Aug 21;9:12183. doi: 10.1038/s41598-019-48257-3 (PMC6704178; doi:10.1038/s41598-019-48257-3)
Supplement: Supplementary file 1 — Supplementary Figures 1 to 6 [file 41598_2019_48257_MOESM1_ESM.pdf]

HIGH DENSITY GENETIC MAPS OF SEASHORE PASPALUM USING GENOTYPING-BY-SEQUENCING AND THEIR RELATIONSHIP TO THE *SORGHUM BICOLOR* GENOME

Peng Qi, Douglas Eudy, James C. Schnable, Jeremy Schmutz, Paul L. Raymer and Katrien M. Devos

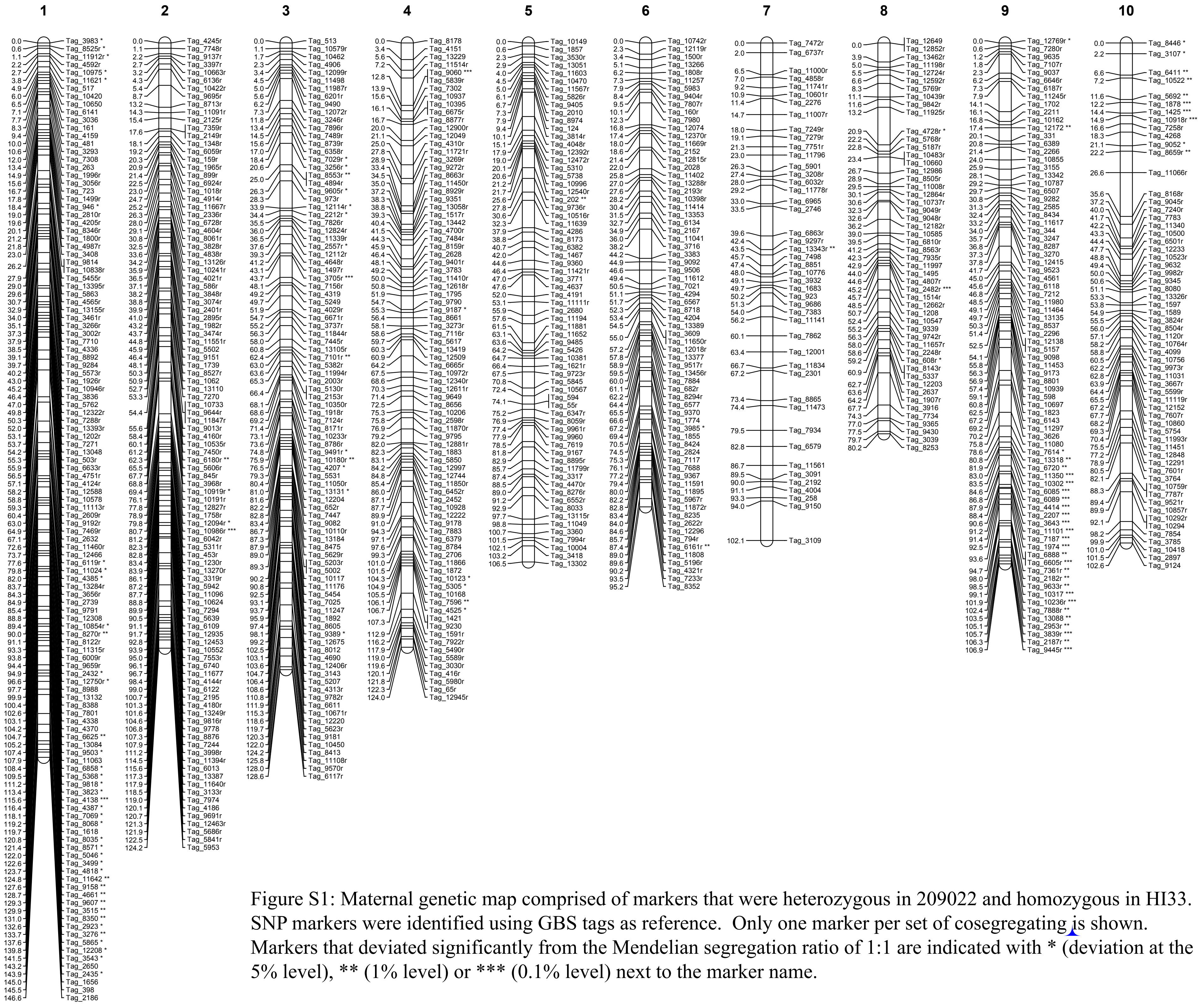

Figure S1: Maternal genetic map comprised of markers that were heterozygous in 209022 and homozygous in HI33. SNP markers were identified using GBS tags as reference. Only one marker per set of cosegregating is shown. Markers that deviated significantly from the Mendelian segregation ratio of 1:1 are indicated with * (deviation at the 5% level), ** (1% level) or *** (0.1% level) next to the marker name.

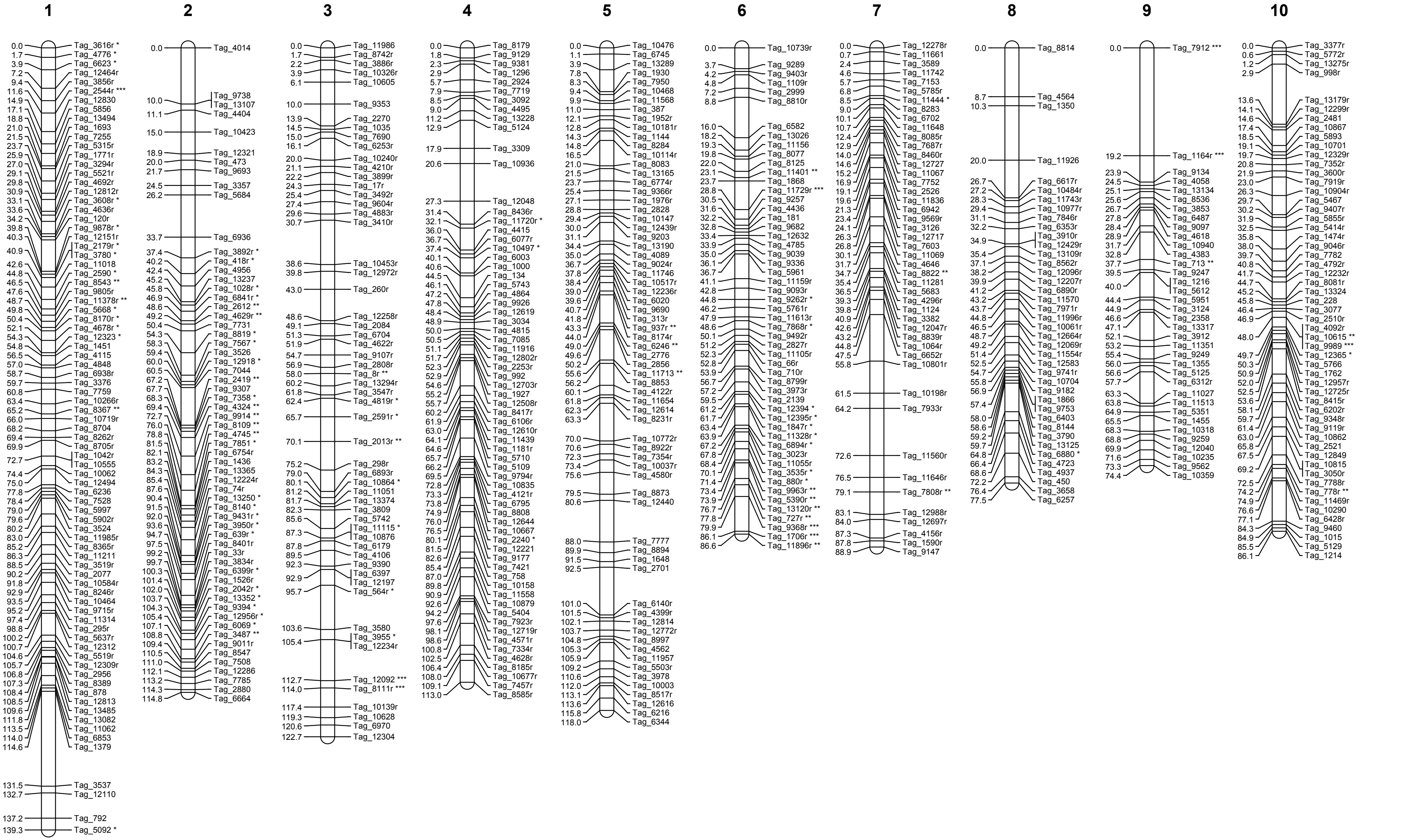

Figure S2: Paternal genetic map comprised of markers that were homozygous in 209022 and heterozygous in HI33. SNP markers were identified using GBS tags as reference. Only one marker per set of cosegregating is shown. Markers that deviated significantly from the Mendelian segregation ratio of 1:1 are indicated with * (deviation at the 5% level), ** (1% level) or *** (0.1% level) next to the marker name.

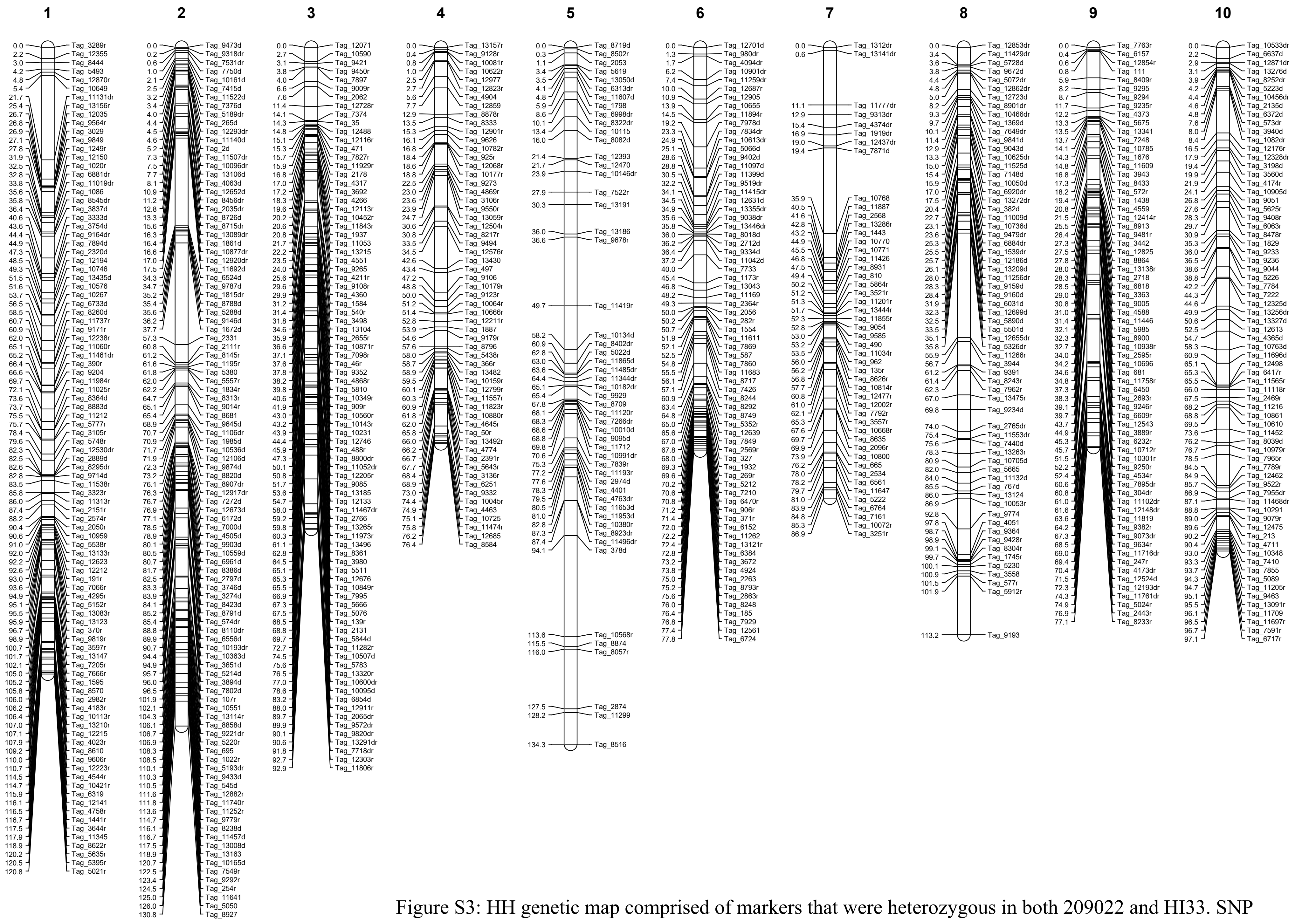

Figure S3: HH genetic map comprised of markers that were heterozygous in both 209022 and HI33. SNP markers were identified using GBS tags as reference. Only one marker per set of cosegregating is shown.

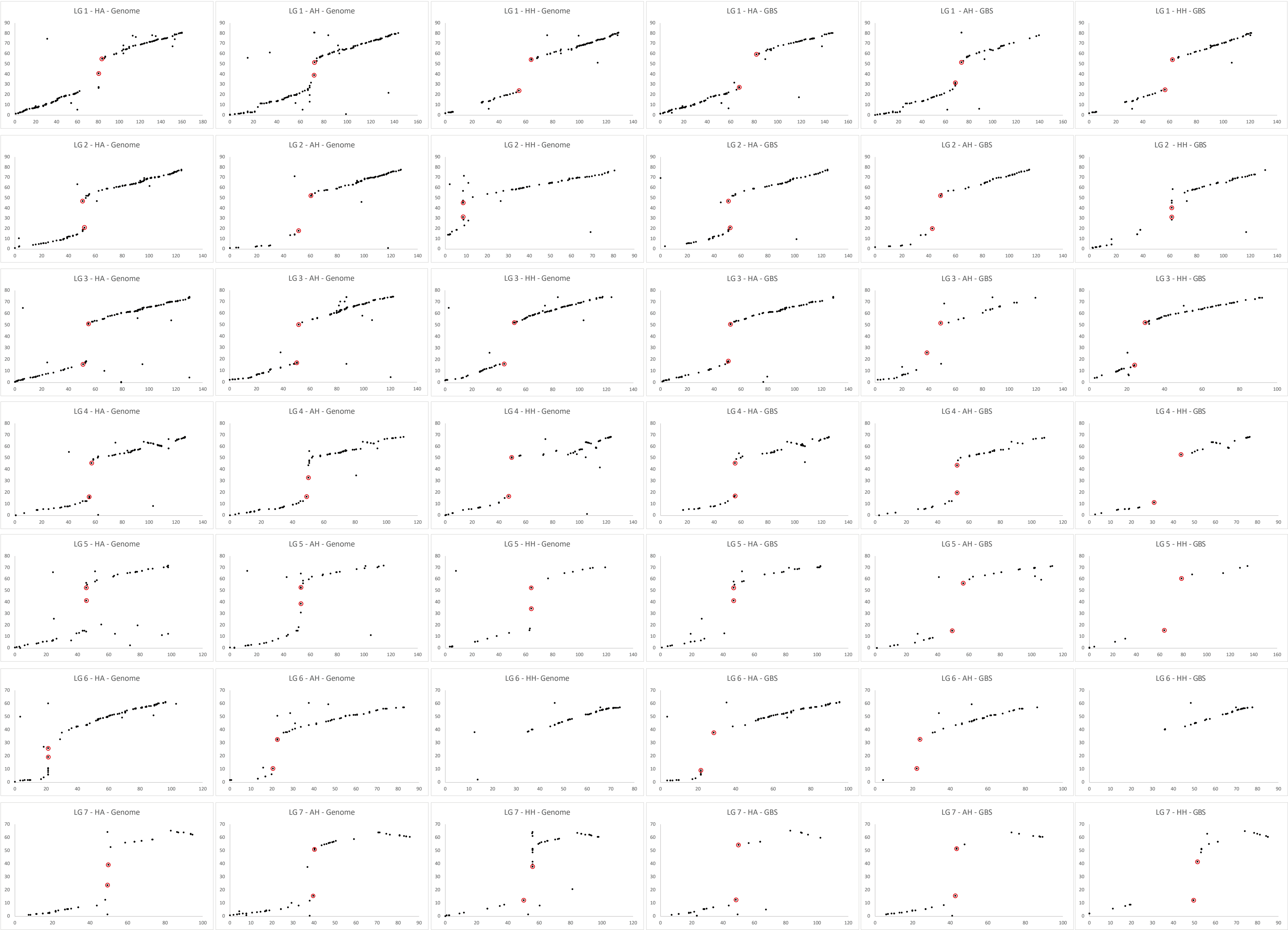
LG 1 - HA - Genome
LG 1 - AH - Genome
LG 1 - HH - Genome
LG 1 - HA - GBS
LG 1 - AH - GBS
LG 1 - HH - GBS
LG 2 - HA - Genome
LG 2 - AH - Genome
LG 2 - HH - Genome
LG 2 - HA - GBS
LG 2 - AH - GBS
LG 2 - HH - GBS
LG 3 - HA - Genome
LG 3 - AH - Genome
LG 3 - HH - Genome
LG 3 - HA - GBS
LG 3 - AH - GBS
LG 3 - HH - GBS
LG 4 - HA - Genome
LG 4 - AH - Genome
LG 4 - HH - Genome
LG 4 - HA - GBS
LG 4 - AH - GBS
LG 4 - HH - GBS
LG 5 - HA - Genome
LG 5 - AH - Genome
LG 5 - HH - Genome
LG 5 - HA - GBS
LG 5 - AH - GBS
LG 5 - HH - GBS
LG 6 - HA - Genome
LG 6 - AH - Genome
LG 6 - HH- Genome
LG 6 - HA - GBS
LG 6 - AH - GBS
LG 6 - HH - GBS
LG 7 - HA - Genome
LG 7 - AH - Genome
LG 7 - HH - Genome
LG 7 - HA - GBS
LG 7 - AH - GBS
LG 7 - HH - GBS

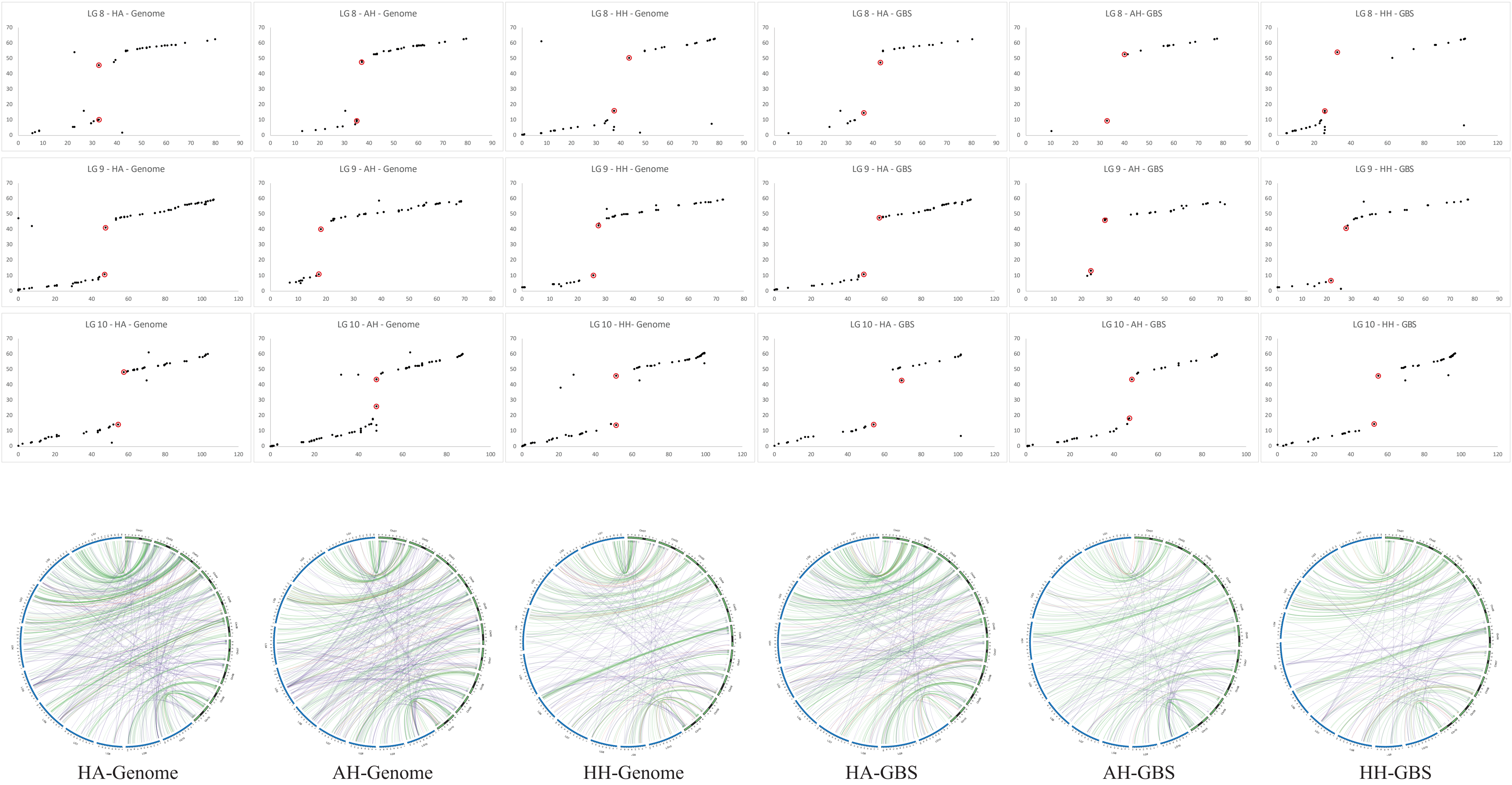

Figure S4: Dot plots and circos diagrams showing the relationship between the seashore paspalum genetic maps (X-axis on dot plots; blue bars labeled as ‘LG’ on circos diagrams) and the sorghum genome sequence (Y-axis on dot plots; green bars labeled as ‘Chr’ on circos diagrams). Green lines on circos diagrams indicate markers that are colinear between the two genomes, red lines indicate markers that are syntenic but not colinear, and purple lines indicate non-syntenic markers. Black bars on the sorghum chromosomes indicate centromere regions. On the dot plots, markers flanking the centromere regions in sorghum are indicated in red.

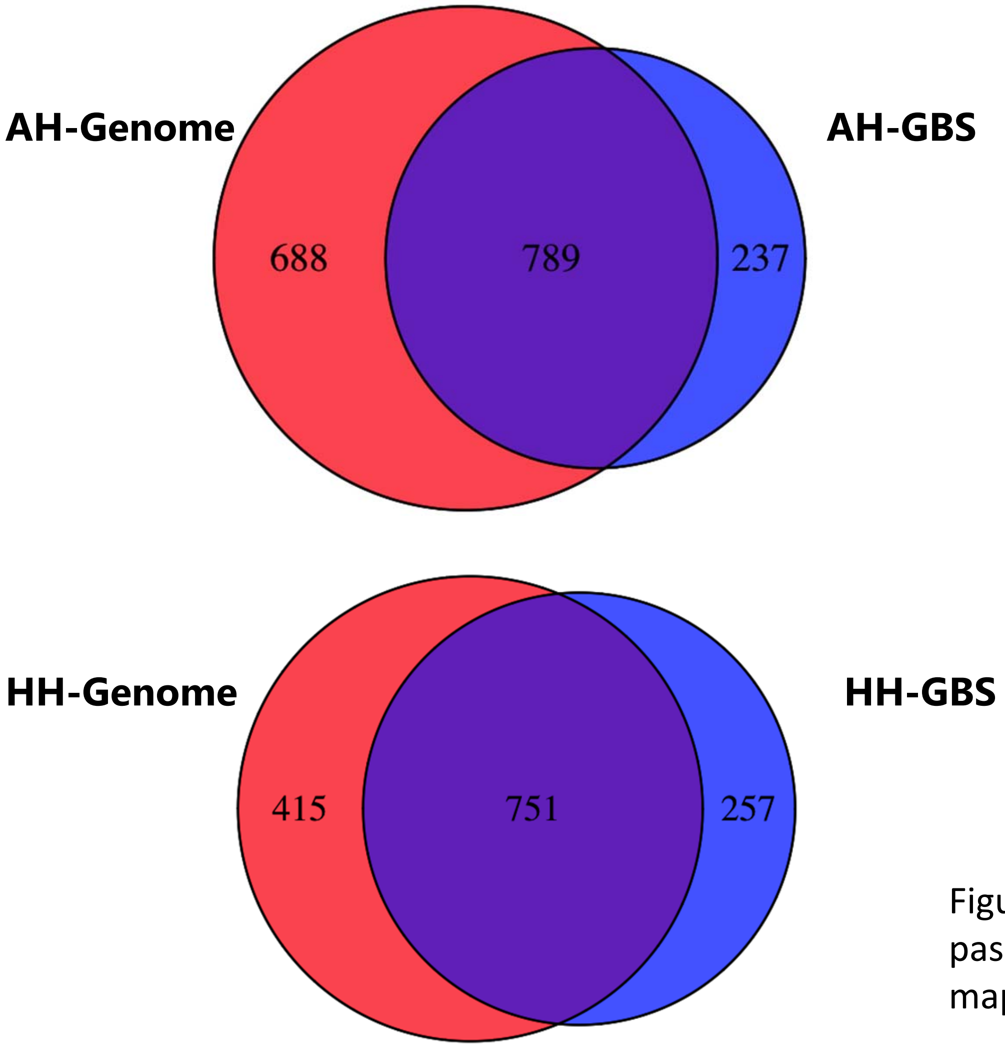

Figure S5: Venn diagrams showing the number of seashore paspalum reference genome scaffolds anchored to the AH maps and HH maps.

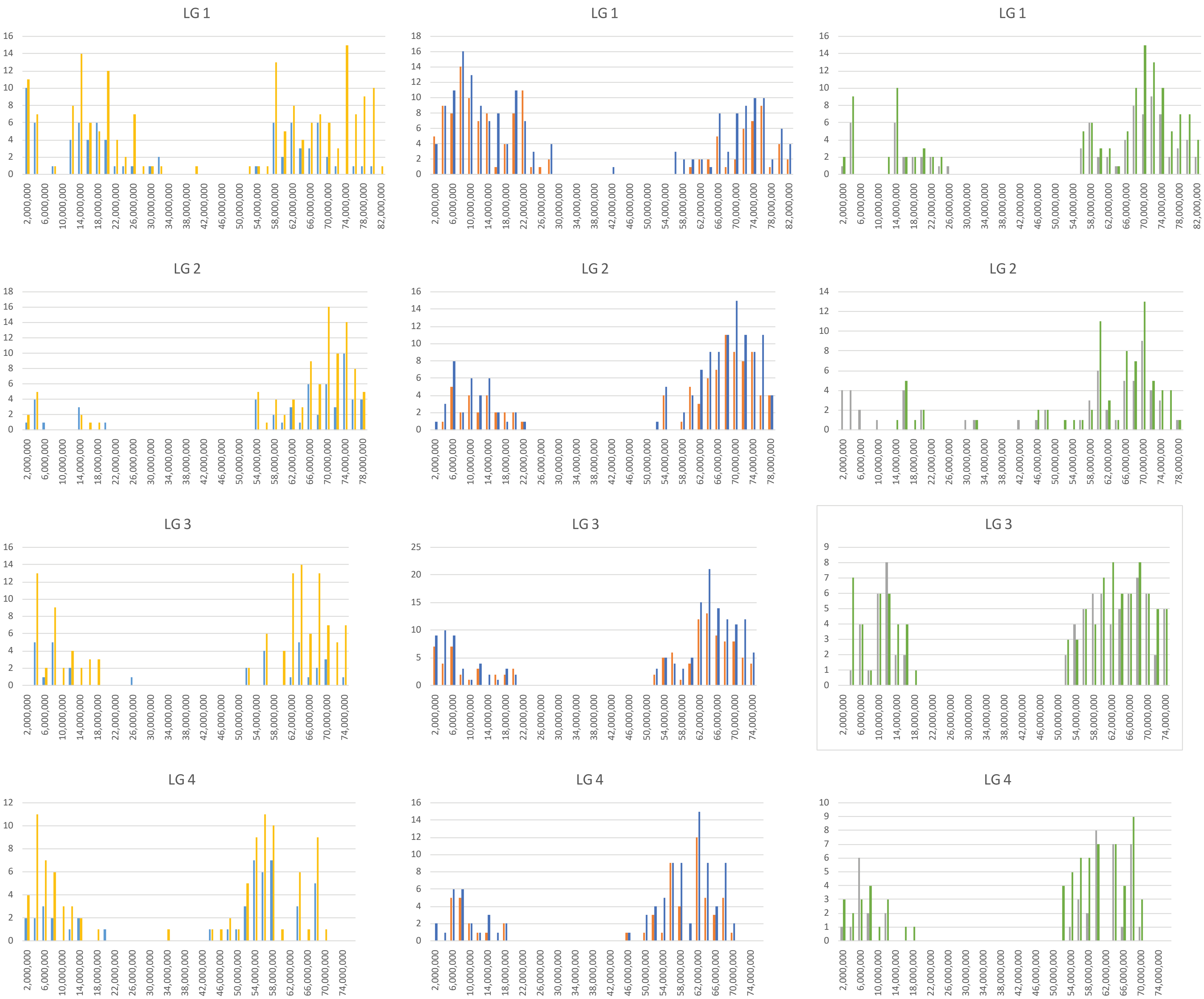
LG 1
LG 1
LG 1
LG 2
LG 2
LG 2
LG 3
LG 3
LG 3
LG 4
LG 4
LG 4

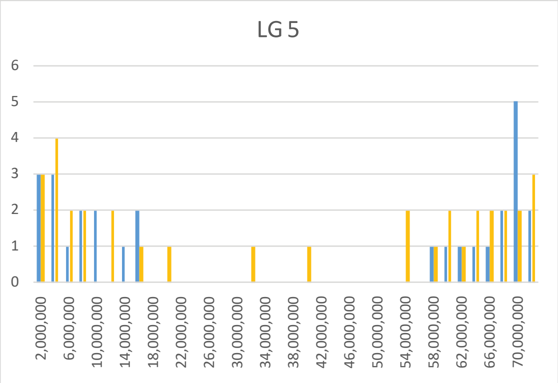
LG 5
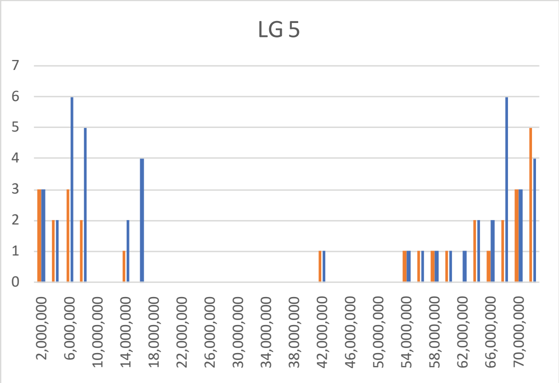
LG 5
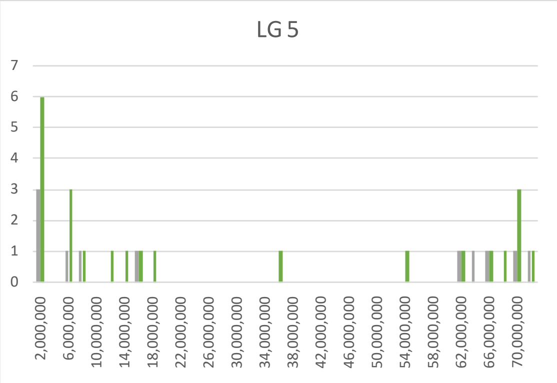
LG 5
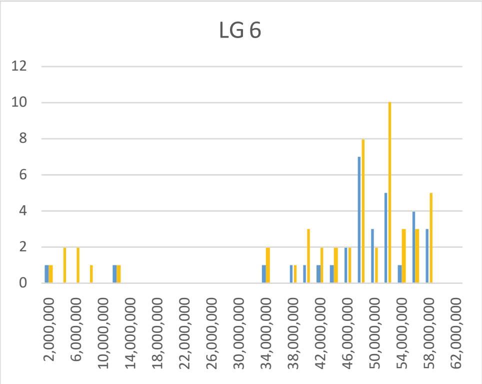
LG 6
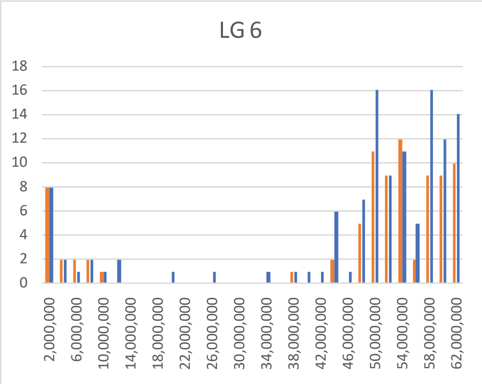
LG 6
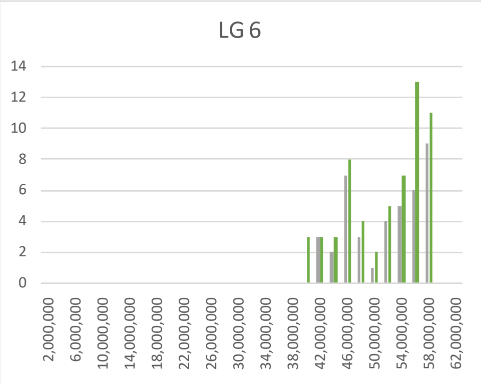
LG 6
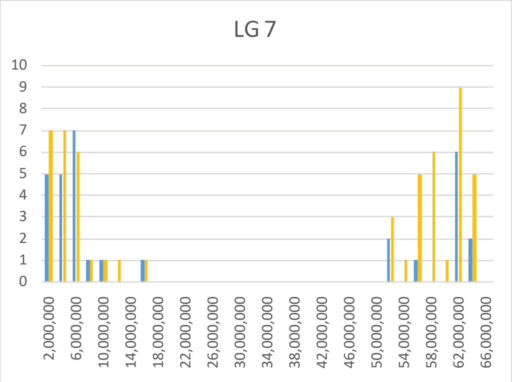
LG 7
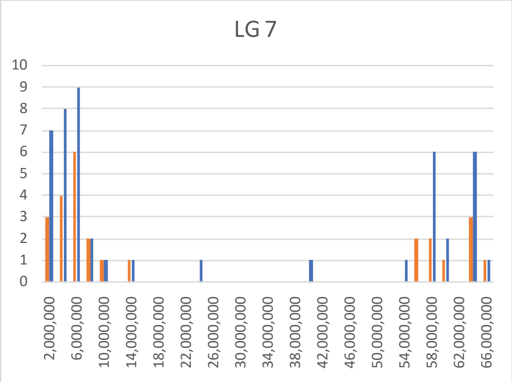
LG 7
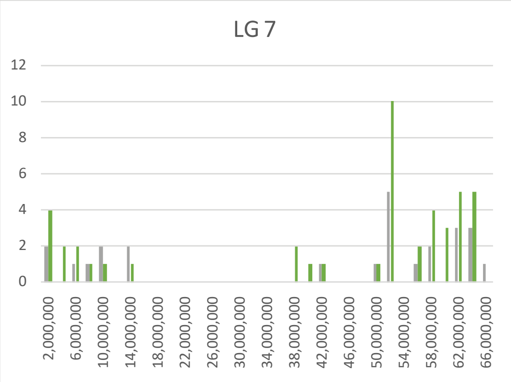
LG 7
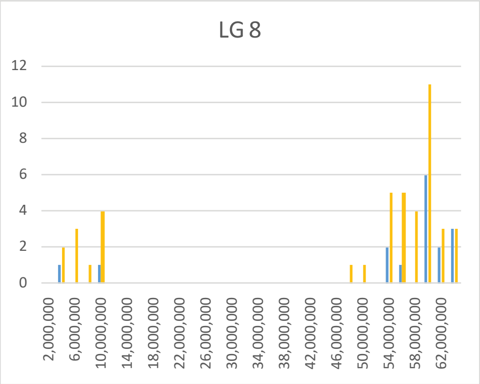
LG 8
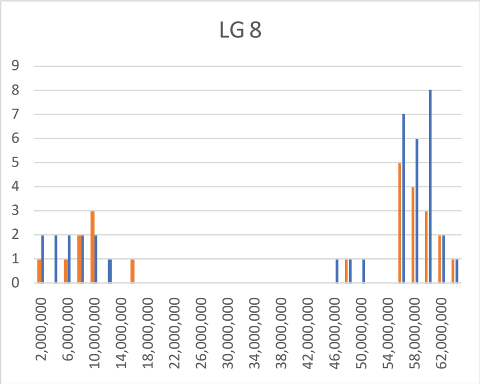
LG 8
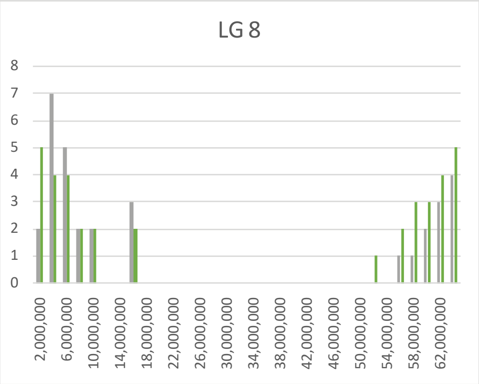
LG 8

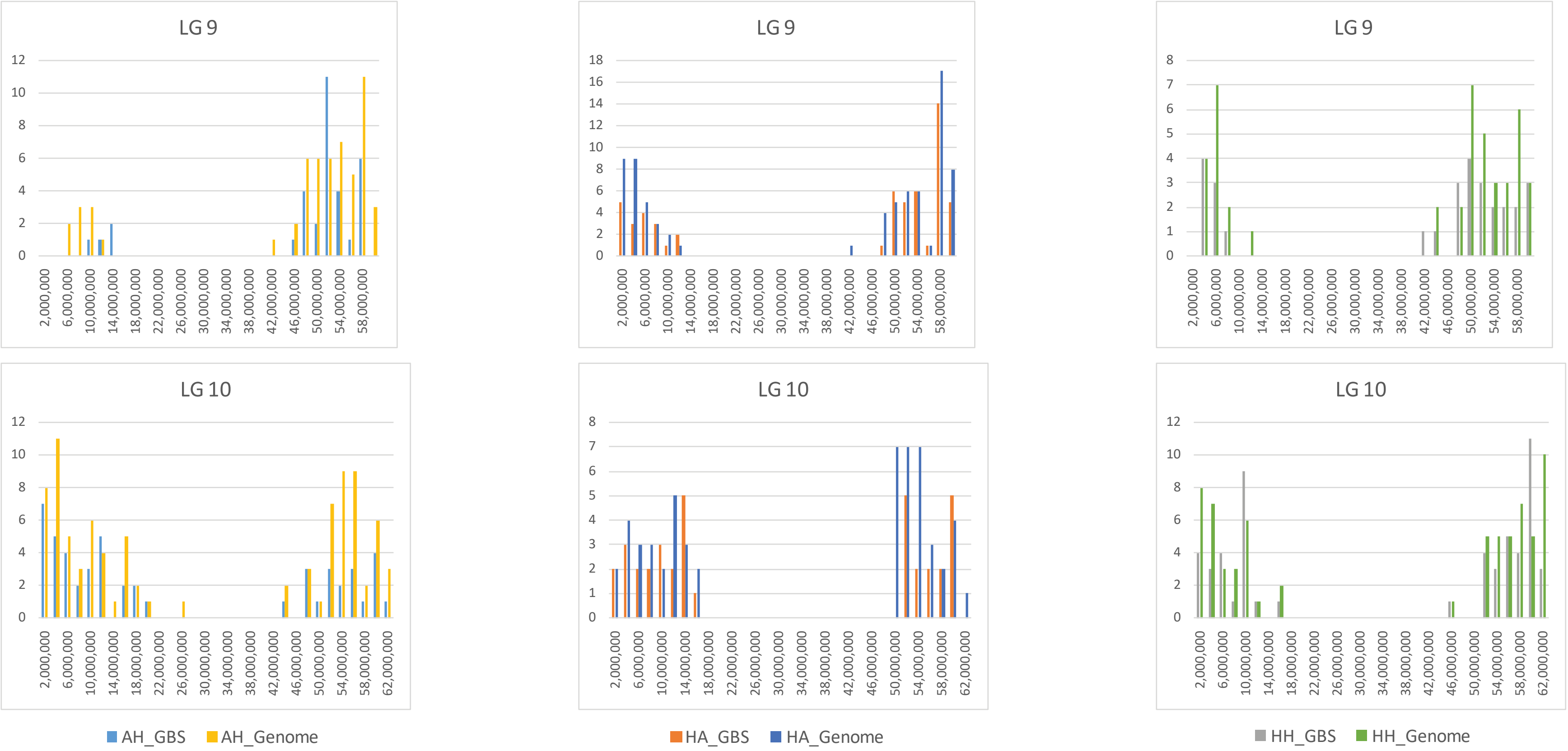

Figure S6: Number of markers mapped (Y-axis) in the seashore paspalum linkage maps in 2 million base pairs windows (X-axis) of the *Sorghum bicolor* genome. Column 1 represents data from the AH maps, column 2 from the HA maps and column 3 from the HH maps.
